# Supplementary material for: Effects of Sodium Selenite Injection on Serum Metabolic Profiles in Women Diagnosed with Breast Cancer-Related Lymphedema—Secondary Analysis of a Randomized Placebo-Controlled Trial Using Global Metabolomics
Source: Nutrients. 2021 Sep 18;13(9):3253. doi: 10.3390/nu13093253 (PMC8470409; doi:10.3390/nu13093253)
Supplement: Supplementary file 1 [file nutrients-13-03253-s001.zip › nutrients-1337185-supplementary.pdf]

Table S1. General and surgical characteristics of participants.

		CTRL (n = 14)	SE (n = 15)	p
**Age (years)**		51.29 ± 14.48 (n = 14)	48.87 ± 11.25 (n = 15)	0.477 [a]
**BMI categories (%)**	**Normal weight**	78.6 (n = 11)	53.3 (n = 8)	0.150 [b]
	**Overweight/Obese**	21.4 (n = 3)	46.7 (n = 7)	
**Post-surgery time (years)**		3.31 ± 2.53 (n = 13)	2.21 ± 1.89 (n = 14)	0.106 [a]

[a]: *Student T*-test was used for comparison of age and the post-surgery time (years) between groups.

[b]: Chi-square test was used for comparison between the composition of normal weight, overweight, and obese according to the subjects' BMI data of groups and the composition of clinical stages according to diagnosis based on clinical observation by physicians at three time points. * $p < 0.05$, ** $p < 0.01$

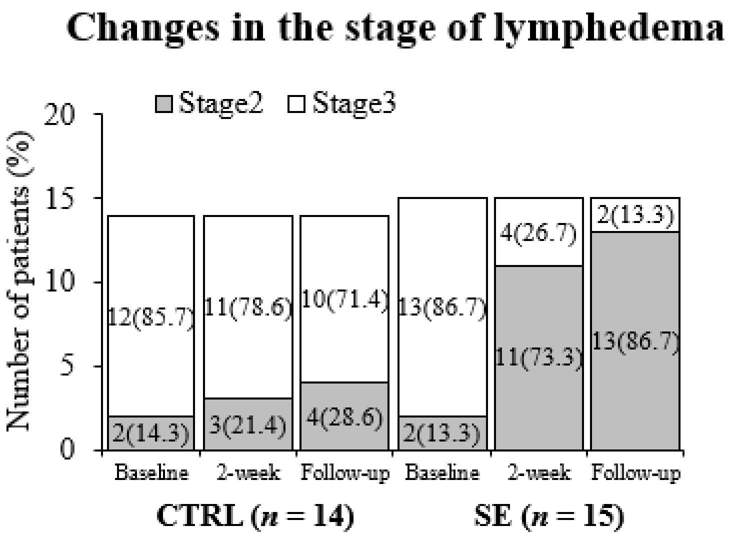

Figure S1. Change of lymphedema stage in CTRL and SE during previous clinical study. CTRL and SE were treated 5 times within 2 weeks, respectively (CTRL, 50mL Normal Saline on IV injection; SE, Sodium selenite 500 μg + 50 mL N/S on IV injection.). Diagnosis was based on clinical observation by physicians at three time points (Baseline, 2-week, Follow-up (6 weeks from baseline)). The numbers in bar represent number of patients in each stage and percentages in each group.

Table S2. Database information and statistical analysis of 107 metabolites significantly altered in SE group than CTRL.

No	HMDB	m/z	RT (min)	Metabolite	VIP	*p*	Log_2FC	Up/ Down	Superpathway	Pathway
1	HMDB11536	357.2407	17.07	MG(0:0/18:1(11Z)/0:0)	4.4689	0.029	1.3895	Up	Lipids	Glycerolipids
2	HMDB09144	786.582	17.88	PE(18:3(6Z-9Z-12Z)/22:6(4Z-7Z-10Z-13Z-16Z-19Z))	3.3005	0.012	3.116	Up	Lipids	Glycerophospholipids
3	HMDB13166	388.2269	15.48	2-Hydroxymyristoylcarnitine	3.0645	0.046	1.457	Up	Lipids	Fatty Acyls
4	HMDB02577	585.3746	6.47	Cholic acid glucuronide	3.0448	0.002	-4.8201	Down	Lipids	Steroids and steroid derivatives
5	HMDB13132	264.2321	12.91	Hydroxyvalerylcarnitine	2.8200	0	-1.5345	Down	Lipids	Fatty Acyls
6	HMDB12350	754.4418	15.14	PS(14:1(9Z)/20:4(5Z-8Z-11Z-14Z))	2.7998	0.001	-2.9647	Down	Lipids	Glycerophospholipids
7	HMDB04185	249.0755	4.38	5-Hydroxyindoleacetylglycine	2.7676	0	1.9269	Up	Amino acid	Carboxylic acids and derivatives
8	HMDB07014	567.4558	21.39	DG(14:0/18:1(11Z)/0:0)	2.7380	0.002	-2.7822	Down	Lipids	Glycerolipids
9	HMDB07007	417.2381	13.21	CPA(18:2(9Z-12Z)/0:0)	2.5804	0.008	-3.8635	Down	Lipids	Fatty Acyls
10	HMDB11559	441.3321	6.17	MG(0:0/24:1(15Z)/0:0)	2.5529	0.007	-4.6508	Down	Lipids	Glycerolipids
11	HMDB03162	599.4456	21.25	7-Methylhypoxanthine	2.5419	0.006	-2.626	Down	Nucleotide	Imidazopyrimidines
12	HMDB00881	206.0845	4.1	Xanthurenic acid	2.5185	0.001	-2.4	Down	Amino acid	Quinolines and derivatives
13	HMDB08871	710.4167	15.29	PE(14:1(9Z)/20:4(5Z-8Z-11Z-14Z))	2.4721	0.005	-2.5286	Down	Lipids	Glycerophospholipids
14	HMDB02224	242.1302	10.42	5-Methyldeoxycytidine	2.4458	0.014	7.5308	Up	Nucleotide	Pyrimidine nucleosides
15	HMDB00288	325.055	0.99	Uridine 5'-monophosphate	2.3893	0.003	2.1487	Up	Nucleotide	Pyrimidine nucleotides
16	HMDB05385	881.7554	20.91	TG(16:0/18:1(9Z)/20:4(5Z-8Z-11Z-14Z))[iso6]	2.3413	0.009	2.6213	Up	Lipids	Glycerolipids
17	HMDB10440	855.6938	21.6	TG(18:1(11Z)/16:0/18:3(9Z-12Z-15Z))[iso6]	2.2902	0.023	-1.9702	Down	Lipids	Glycerolipids
18	HMDB05369	859.7751	21.33	TG(16:0/18:0/18:2(9Z-12Z))[iso6]	2.2891	0.012	1.7635	Up	Lipids	Glycerolipids
19	HMDB11491	536.4373	21.39	LysoPE(0:0/22:1(13Z))	2.2557	0.02	-2.587	Down	Lipids	Glycerophospholipids
20	HMDB06880	390.1077	12.63	Acetyl adenylate	2.2229	0.001	1.996	Up	Lipids	Steroids and steroid derivatives
21	HMDB01084	128.0762	4.4	D-1-Piperideine-2-carboxylic acid	2.2184	0.029	-0.77889	Down	Amino acid	Pyridines and derivatives
22	HMDB09639	838.5585	18.65	PE(22:5(4Z-7Z-10Z-13Z-16Z)/22:6(4Z-7Z-10Z-13Z-16Z-19Z))	2.2174	0.004	1.9742	Up	Lipids	Glycerophospholipids
23	HMDB01539	203.1387	8.45	Dimethyl-L-arginine	2.1886	0.041	3.3745	Up	Amino acid	Carboxylic acids and derivatives

No	HMDB	m/z	RT (min)	Metabolite	VIP	*p*	Log_2FC	Up/ Down	Superpathway	Pathway
24	HMDB01866	185.0645	10.98	3-4-Dihydroxymandelic acid	2.1613	0.033	1.7765	Up	-	Phenols
25	HMDB00735	210.0757	4.59	Hydroxyphenylacetylglycine	2.1272	0.004	2.6837	Up	Amino acid	Carboxylic acids and derivatives
26	HMDB01128	230.1385	9.69	5-Phosphoribosylamine	2.1257	0.036	2.5874	Up	Cofactor/vitamin	Organooxygen compounds
27	HMDB01474	251.1155	14.81	3-4-Dihydroxyphenylglycol O-sulfate	2.0836	0.01	-0.75711	Down	-	Organic sulfuric acids and derivatives
28	HMDB07019	561.3642	8.18	DG(14:0/18:4(6Z-9Z-12Z-15Z)/0:0)	2.0609	0.048	-2.4867	Down	Lipids	Fatty Acyls
29	HMDB02649	121.0319	4.49	Erythrose	2.0290	0.029	-0.2744	Down	Carbohydrate	Organooxygen compounds
30	HMDB00708	450.2746	14.03	Glycoursodeoxycholic acid	2.0142	0.043	-1.0995	Down	Lipids	Steroids and steroid derivatives
31	HMDB11618	289.2472	14.29	All-trans-13-14-dihydroretinol	1.9974	0.016	1.0468	Up	Lipids	Prenol lipids
32	HMDB05767	255.1105	12.35	Homoanserine	1.9952	0.002	3.2237	Up	Amino acid	Peptidomimetics
33	HMDB13164	360.2378	7.32	2-Hydroxylauroylcarnitine	1.9946	0.041	-1.6975	Down	Lipids	Fatty Acyls
34	HMDB00905	332.1364	14.12	Deoxyadenosine monophosphate	1.9773	0.015	0.64989	Up	Nucleotide	Purine nucleotides
35	HMDB00101	252.1203	9.66	Deoxyadenosine	1.9732	0.046	2.0023	Up	Nucleotide	Purine nucleosides
36	HMDB01409	309.1205	13.43	dUMP	1.9453	0.004	1.1322	Up	Nucleotide	Pyrimidine nucleotides
37	HMDB10477	881.7188	20.79	TG(18:2(9Z-12Z)/18:0/18:3(9Z-12Z-15Z))[iso6]	1.9434	0.02	-1.3302	Down	Lipids	Glycerolipids
38	HMDB02096	204.1227	6.33	3-Indolebutyric acid	1.9179	0.033	2.2824	Up	-	Indoles and derivatives
39	HMDB00232	168.0133	0.62	Quinolinic acid	1.8760	0.041	2.3101	Up	-	Pyridines and derivatives
40	HMDB06954	182.115	7.33	2-Methyl-3-hydroxy-5-formylpyridine-4-carboxylate	1.8616	0.046	2.513	Up	Carbohydrate	Pyridines and derivatives
41	HMDB03533	435.3806	18.48	3a-7a-12a-Trihydroxy-5b-cholestan-26-al	1.8302	0.014	1.4813	Up	Lipids	Steroids and steroid derivatives
42	HMDB00785	352.0814	2.48	N-Acetyl-7-O-acetylneuraminic acid	1.8268	0.025	1.6445	Up	Carbohydrate	Organooxygen compounds
43	HMDB00963	261.1006	10.34	5-Methylthioribose 1-phosphate	1.7842	0.006	1.1691	Up	Carbohydrate	Organooxygen compounds
44	HMDB10391	550.3867	18.01	LysoPC(20:1(11Z))	1.7725	0.041	1.0671	Up	Lipids	Glycerophospholipids
45	HMDB10313	315.1005	7.17	1-Salicylate glucuronide	1.7692	0.014	-0.77416	Down	-	Organooxygen compounds
46	HMDB11685	297.1694	16.16	DHAP(8:0)	1.7639	0.026	-0.6451	Down	Lipids	Organooxygen compounds
47	HMDB00894	144.1381	13.57	Vinylacetylglycine	1.7599	0.001	-0.66845	Down	Lipids	Carboxylic acids and derivatives

No	HMDB	m/z	RT (min)	Metabolite	VIP	*p*	Log_2FC	Up/ Down	Superpathway	Pathway
48	HMDB03466	179.081	8.83	L-Gulonolactone	1.7337	0.004	0.88255	Up	Cofactor/vitamin	Lactones
49	HMDB00715	190.0464	4.39	Kynurenic acid	1.7108	0.014	0.91881	Up	Amino acid	Quinolines and derivatives
50	HMDB13326	342.173	13.36	trans-2-Dodecenoylcarnitine	1.6927	0.009	-0.90037	Down	Lipids	Fatty Acyls
51	HMDB09410	850.595	18.93	PE(20:4(5Z-8Z-11Z-14Z)/24:1(15Z))	1.6908	0.018	1.8878	Up	Lipids	Glycerophospholipids
52	HMDB01439	367.1495	5.8	Phosphoribosyl formamidocarboxamide	1.6689	0.002	0.9822	Up	Nucleotide	Imidazole ribonucleosides and ribonucleotides
53	HMDB08284	870.7544	21.42	PC(20:0/22:2(13Z-16Z))	1.6601	0.026	1.2875	Up	Lipids	Glycerophospholipids
54	HMDB12144	290.1342	1.88	2-Amino-4-oxo-6-(1'-2'-3'-trihydroxypropyl)-diquinoid-7-8-dihydroxypterin	1.6571	0.014	-0.82606	Down	-	Pteridines and derivatives
55	HMDB00721	173.0287	20.76	Glycylproline	1.6468	0.046	-0.67631	Down	Amino acid	Carboxylic acids and derivatives
56	HMDB00913	213.1093	11.01	Vanillactic acid	1.6282	0.012	0.88265	Up	-	Phenylpropanoic acids
57	HMDB02282	366.1848	17.9	2-(acetylamino)-1-5-anhydro-2-deoxy-4-O-b-D-galactopyranosyl-D-arabino-Hex-1-enitol	1.6160	0.026	-0.58711	Down	Carbohydrate	Organooxygen compounds
58	HMDB12289	172.0061	6.66	Tetrahydrodipicolinate	1.6074	0.004	0.93637	Up	Amino acid	Carboxylic acids and derivatives
59	HMDB00044	176.97	21.70	Ascorbic acid	1.60	0.023	0.753	Up	Cofactor/vitamin	
60	HMDB01547	347.1598	9.27	Corticosterone	1.5969	0.008	0.93494	Up	Lipids	Steroids and steroid derivatives
61	HMDB10390	552.329	15.98	LysoPC(20:0)	1.5797	0.033	1.2238	Up	Lipids	Glycerophospholipids
62	HMDB00439	170.0921	2.64	2-Furoylglycine	1.5537	0.041	1.0786	Up	Amino acid	Carboxylic acids and derivatives
63	HMDB12281	375.1482	11.06	Portulacaxanthin II	1.5338	0.041	0.95982	Up	Amino acid	Carboxylic acids and derivatives
64	HMDB13330	386.2899	15.12	3-Hydroxy-cis-5-tetradecenoylcarnitine	1.5273	0.029	-0.8781	Down	-	-
65	HMDB11678	408.3106	16.48	Geranylgeranylcysteine	1.5210	0.029	-1.0844	Down	Lipids	Prenol lipids
66	HMDB00045	348.1227	15.28	Adenosine monophosphate	1.5158	0.046	-0.60856	Down	Nucleotide	Purine nucleotides
67	HMDB09045	790.5586	18.22	PE(18:1(11Z)/22:6(4Z-7Z-10Z-13Z-16Z-19Z))	1.5148	0.037	1.3745	Up	Lipids	Glycerophospholipids
68	HMDB01185	400.1862	8.38	S-Adenosylmethionine	1.5121	0.041	1.2659	Up	Nucleotide	5'-deoxyribonucleosides
69	HMDB05773	611.2859	16.62	Endomorphin-1	1.5021	0.029	-0.83609	Down	Amino acid	Carboxylic acids and derivatives
70	HMDB00497	247.0849	10.34	5-6-Dihydrouridine	1.4884	0.018	0.7884	Up	Nucleotide	Organooxygen compounds

No	HMDB	m/z	RT (min)	Metabolite	VIP	*p*	Log_2FC	Up/ Down	Superpathway	Pathway
71	HMDB00982	258.2062	15.09	5-Methylcytidine	1.4794	0.016	0.79837	Up	Nucleotide	Pyrimidine nucleosides
72	HMDB01000	389.0949	12.03	dUDP	1.4553	0.002	1.0291	Up	Nucleotide	Pyrimidine nucleotides
73	HMDB12329	301.1771	15.9	4-Oxoretinol	1.4414	0.018	0.85594	Up	Lipids	Prenol lipids
74	HMDB11639	212.0662	12.08	Topaquinone	1.4311	0.046	-1.6208	Down	Amino acid	Carboxylic acids and derivatives
75	HMDB01431	169.06	9.43	Pyridoxamine	1.42	0.029	1.779	Up	Cofactor/vitamin	
76	HMDB00194	241.18	13.86	Anserine	1.41	0.029	0.725	Up	Amino acid	
77	HMDB06555	333.144	11.58	dIMP	1.3904	0.006	1.3349	Up	Nucleotide	Purine nucleotides
78	HMDB09138	798.599	20.25	PE(18:3(6Z-9Z-12Z)/22:0)	1.3891	0.023	-0.65815	Down	Lipids	Glycerophospholipids
79	HMDB07105	591.459	18.1	DG(16:0/18:3(9Z-12Z-15Z)/0:0)	1.3822	0.037	0.74716	Up	Lipids	Fatty Acyls
80	HMDB05085	364.3206	18.54	Leukotriene B4 dimethylamide	1.3799	0.029	0.64707	Up	Lipids	Fatty Acyls
81	HMDB00235	266.1385	6.77	Thiamine	1.3687	0.041	-1.2446	Down	Cofactor/vitamin	Diazines
82	HMDB07021	595.4921	18.18	DG(14:0/20:1(11Z)/0:0)	1.3629	0.029	1.0145	Up	Lipids	Glycerolipids
83	HMDB07072	581.4483	19.03	DG(15:0/18:1(11Z)/0:0)	1.3568	0.046	0.75851	Up	Lipids	Glycerolipids
84	HMDB04985	262.1282	1.66	Aspartylysine	1.3474	0.037	0.81696	Up	Amino acid	Carboxylic acids and derivatives
85	HMDB01304	321.2364	14.33	QH2	1.3411	0.046	1.1561	Up	Lipids	Prenol lipids
86	HMDB01117	403.1105	12.03	4-Phosphopantothenoylcysteine	1.3262	0.003	0.94931	Up	Cofactor/vitamin	Peptidomimetics
87	HMDB00015	347.16	11.8	Cortexolone	1.3231	0.01	0.68451	Up	Lipids	Steroids and steroid derivatives
88	HMDB03364	109.1013	10.34	Quinone	1.3207	0.046	0.83975	Up	-	Organooxygen compounds
89	HMDB07031	617.4741	18.19	DG(14:0/22:4(7Z-10Z-13Z-16Z)/0:0)	1.3126	0.041	0.94109	Up	Lipids	Glycerolipids
90	HMDB03259	365.284	16.84	Dihydrocortisol	1.3056	0.046	1.0656	Up	Lipids	Steroids and steroid derivatives
91	HMDB04821	217.0381	5.53	Bisnorbiotin	1.2942	0.037	0.54424	Up	Cofactor/vitamin	Carboxylic acids and derivatives
92	HMDB11154	395.2914	18.79	LPA(P-16:0e/0:0)	1.2929	0.041	1.0244	Up	Lipids	Glycerophospholipids
93	HMDB03332	265.0228	1.13	3-Methoxy-4-Hydroxyphenylglycol sulfate	1.2565	0.018	1.1664	Up	Benzenoids	Phenols
94	HMDB11112	279.0749	2.5	N1-(alpha-D-ribosyl)-5-6-dimethyl-benzimidazole	1.2180	0.023	0.73459	Up	Nucleotide	Benzimidazole ribonucleosides and ribonucleotides

No	HMDB	m/z	RT (min)	Metabolite	VIP	*p*	Log_2FC	Up/ Down	Superpathway	Pathway
95	HMDB11544	383.2789	16.74	MG(0:0/20:2(11Z-14Z)/0:0)	1.2029	0.008	0.65465	Up	Lipids	Endocannabinoids
96	HMDB02664	351.1547	10.75	Prostaglandin E3	1.1943	0.002	1.1207	Up	Lipids	Fatty Acyls
97	HMDB02432	143.0677	4.76	Sumiki's acid	1.1879	0.029	0.65003	Up	-	Furans
98	HMDB01200	237.0643	6.26	N'-Formylkynurenine	1.1774	0.037	0.57802	Up	Amino acid	Organooxygen compounds
99	HMDB13331	368.2791	15.13	3- 5-Tetradecadiencarnitine	1.1769	0.041	-0.31852	Down	-	-
100	HMDB02089	288.2165	12.04	N-Ribosylhistidine	1.1739	0.037	-0.57405	Down	Amino acid	Carboxylic acids and derivatives
101	HMDB00038	240.1956	14.74	Dihydrobiopterin	1.1724	0.046	0.4855	Up	-	Pteridines and derivatives
102	HMDB06275	234.0182	6.26	Dopamine 3-O-sulfate	1.1352	0.018	0.59275	Up	Nucleotide	Organic sulfuric acids and derivatives
103	HMDB02721	283.1026	12.03	1-Methylinosine	1.1095	0.004	0.90296	Up	Amino acid	Purine nucleosides
104	HMDB00732	225.1063	12.38	Hydroxykynurenine	1.0693	0.026	0.61469	Up	Nucleotide	Organooxygen compounds
105	HMDB00229	335.1234	11.43	Nicotinamide ribotide	1.0591	0.018	1.0223	Up	Cofactor/vitamin	Pyridine nucleotides
106	HMDB06226	417.3357	15.82	24,25-Dihydroxyvitamin D_3	1.0335	0.037	0.79589	Up	Amino acid	Steroids and steroid derivatives
107	HMDB00734	188.0706	9.62	Indoleacrylic acid	1.0100	0.026	0.69922	Up	-	Indoles and derivatives

Metabolites and their superpathways and pathways were identified on the basis of accurate mass data, retention time (RT), experimental MS/MS spectra, and library MS/MS spectra (HMDB, MyCompoundID, and KEGG). A total of 107 metabolites were selected among the meaningful metabolites (n = 137, VIP > 1.0, p < 0.05) after excluding drugs, xenobiotics, and duplicates (n = 30).

Table S3. Summary of pathway analysis.

Pathway Name	Impact	FDR	*p*	-log(p)	Details
Pyrimidine metabolism	0.232	0.003	0.0001	3.9595	KEGG, SMP
Glycerophospholipid metabolism	0.216	0.024	0.0058	2.2386	KEGG
Purine metabolism	0.182	0.006	0.0004	3.3605	KEGG, SMP
Pantothenate and CoA biosynthesis	0.179	0.040	0.0184	1.7357	KEGG, SMP
Vitamin B6 metabolism	0.078	0.043	0.0267	1.5732	KEGG, SMP
Cysteine and methionine metabolism	0.060	0.036	0.0119	1.9244	KEGG, SMP, SMP
Histidine metabolism	0.049	0.043	0.0300	1.5233	KEGG, SMP
Steroid hormone biosynthesis	0.045	0.024	0.0061	2.2175	KEGG, SMP
Primary bile acid biosynthesis	0.034	0.036	0.0137	1.8624	KEGG, SMP
Nicotinate and nicotinamide metabolism	0.032	0.047	0.0341	1.4679	KEGG, SMP
Folate biosynthesis	0.021	0.052	0.0448	1.3487	KEGG, SMP
Glycerolipid metabolism	0.014	0.036	0.0138	1.8600	KEGG, SMP
Tyrosine metabolism	0.007	0.052	0.0460	1.3371	KEGG, SMP, SMP
Glycosylphosphatidylinositol (GPI)-anchor biosynthesis	0.004	0.052	0.0441	1.3553	KEGG
Ascorbate and aldarate metabolism	0.000	0.021	0.0030	2.5290	KEGG
Pyruvate metabolism	0.000	0.021	0.0032	2.4959	KEGG, SMP
Retinol metabolism	0.000	0.024	0.0064	2.1958	KEGG, SMP
Alanine, aspartate and glutamate metabolism	0.000	0.040	0.0180	1.7443	KEGG, SMP
Arachidonic acid metabolism	0.000	0.043	0.0286	1.5436	KEGG, SMP
Linoleic acid metabolism	0.000	0.043	0.0286	1.5436	KEGG, SMP
alpha-Linolenic acid metabolism	0.000	0.043	0.0286	1.5436	KEGG, SMP
beta-Alanine metabolism	0.000	0.043	0.0300	1.5233	KEGG, SMP
Ether lipid metabolism	0.000	0.052	0.0412	1.3856	KEGG
Steroid biosynthesis	0.000	0.074	0.0687	1.1628	KEGG
Thiamine metabolism	0.000	0.109	0.1049	0.9791	KEGG, SMP
Arginine and proline metabolism	0.000	0.110	0.1103	0.9575	KEGG, SMP

Pathway analysis were analyzed based on 107 identified metabolites by Metaboanalyst 4.0. This list is arranged in the order of high impact value, which were considered as more significantly relevant pathway.

Table S4. Significant metabolites associated with whole blood Se level (WBSe) or arm ECW/SW ratio over multiple time point.

Model	Factor	Identification	Estimate	*p*	S.E.	Df	t-value
1	Arm ECW/SW ratio	alpha-Ribazole	-1053.5	0.034	482.509	45.077	-2.183
		Xanthurenic acid	962.0	0.038	452.852	52.201	2.124
		Hydroxyphenylacetylglycine	-4849.8	0.006	1670.598	44.966	-2.903
		2-Methyl-3-hydroxy-5-formylpyridine-4-carboxylate	-18577.1	0.031	8401.430	54.000	-2.211
		24,25-Dihydroxyvitamin D_3	5959.0	0.035	2739.532	46.912	2.175
2	WBSe	Xanthurenic acid	-0.61	0.012	0.233	53.253	-2.595

Linear mixed effect regression analysis (LMM) was used to determine metabolites which were significantly related with both whole blood Se level and the ECW/SW ratio among 107 metabolites over time (Baseline and 2-week). Subjects and time were applied as random effect and fixed effect in the statistic models, respectively. S.E.; standard error, df; degree of freedom
